# The complete chloroplast genome of *Parnassia wightiana* (Celastraceae)

**DOI:** 10.1080/23802359.2019.1688700

**Published:** 2019-11-13

**Authors:** Jianfang Li, Qian Yang, Bei Xu, Zhan-Lin Liu

**Affiliations:** Key Laboratory of Resource Biology and Biotechnology in Western China (Ministry of Education), College of Life Sciences, Northwest University, Xi’an, PR China

**Keywords:** *Parnassia wightiana*, Celastraceae, plastome, phylogeny

## Abstract

The infrageneric relationships of the genus *Parnassia* were not clearly identified due to limited available genetic data. In this study, we presented the complete chloroplast genome of *Parnassia wightiana* for future phylogenetic study. The plastome of *P. wightiana* was 152,043 bp in length, with a large single-copy region (LSC) of 82,737 bp and a small single-copy region (SSC) of 19,030 bp, separated by a pair of inverted repeat regions (IRs) of 25,138 bp. It contained 134 genes, including 87 protein-coding genes, 37 tRNA genes, 8 rRNA genes, and 2 pseudogenes. The overall GC content was 37.1%, while the corresponding values in the LSC, SSC, and IR region were 35.0%, 30.8%, and 42.9%, respectively. The phylogenetic analysis supported the monophyly of *Parnassia*.

The genus *Parnassia* Linnaeus includes 50–70 species occurring in arctic and alpine habitats of the northern hemisphere (Wu and Raven 2001). The infrageneric taxonomic treatment of Parnassia has been unsolved for the limited morphological variation and the restricted geographic distribution of many species (Yang et al. 2012). The rapid development of chloroplast genome sequences provides a great amount of genetic information for species identification and phylogenomic study of the highly diverse lineages. To date, only two *Parnassia* plastomes are reported (Xia et al. 2018). These two species (*P. brevistyla* and *P. trinervis*) are locally distributed in Western China. In this study, we determined the plastome of *Parnassia wightiana*, a widely distributed herb in Central and Western China, Bhutan, North India, Nepal, and North Thailand, to provide additional effective data for the phylogenetic study of *Parnassia* in future.

Fresh leaves of *P. wightiana* were collected from Chongqing of China (N30.23° E108.40°). The voucher (2018LIU0628) was deposited at the Evolutionary Botany Laboratory (EBL), Northwest University. The procedure of DNA isolation, genome sequencing and data processing follow the previous study (Peng et al. 2019). The complete chloroplast genome was annotated with *P. brevistyla* (MG792145) as reference and has been submitted to GenBank with the accession number of MN398191.

The plastome of *P. wightiana* is 152,043 bp in length, with a large single-copy region (LSC) of 82,737 bp, a small single-copy region (SSC) of 19,030 bp, and a pair of inverted repeat regions (IRs) of 25,138 bp. It contains 134 genes, including 87 protein-coding genes, 37 tRNA genes, 8 rRNA genes, and 2 pseudogenes (*ycf*1*, rps*12). The pseudogene *ycf*1 is located in the boundary of IRb and SSC region and the pseudogene *rps*12 is located in IRa region. Eighteen genes are duplicated in the IRs, containing seven protein-coding genes (*rps*19, *rpl*2, *rpl*23, *ycf*2, *ndh*B, *rps*7, and *ycf*15), seven tRNA genes (*trn*I-CAU, *trn*L-CAA, *trn*V-GAC, *trn*I-GAU, *trn*A-UGC, *trn*R-ACG, and *trn*N-GUU), and four rRNA genes (*rrn*16S, *rrn*23S, *rrn*4.5S, and *rrn*5S). Among the annotated genes, 12 (*atp*F, *rpo*C1, *pet*B, *pet*D, *rpl*2, *ndh*B, *ndh*A, *trn*K-UUU, *trn*L-UAA, *trn*V-UAC, *trn*I-GAU, and *trn*A-UGC) contain a single intron and three (*ycf*3, *rps*12, *clp*P) have two introns. The overall GC content is 37.1%, while the corresponding values in the LSC, SSC, and IR region are 35.0%, 30.8%, and 42.9%, respectively.

Due to limited data available for the family Celastraceae, 19 plastomes from representative species in Fabids clade were used to analyze the phylogenetic position of *P. wightiana* with two species (*Viviania marifolia* and *Erodium chrysanthum*) in Malvids as the outgroup. The phylogenetic tree constructed by the maximum likelihood method supported that the genus *Parnassia* was monophyletic and *P. wightiana* was closely related to *P. brevistyla* (Figure 1). The relationships among taxa at the order level were consistent with the results of the APG IV system (Angiosperm Phylogeny Group 2016).

**Figure 1.**
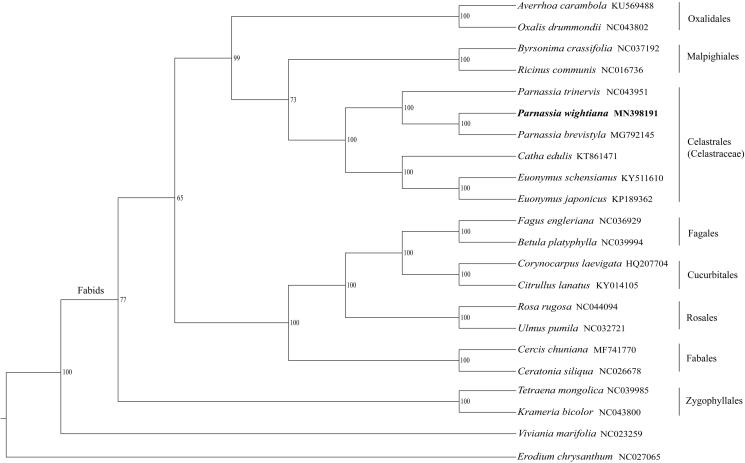
The phylogenetic tree of representatives in the clade of Fabids constructed by maximum likelihood method using the complete chloroplast genome sequences. The bootstrap values based on 1000 replicates were labelled beside the branches.
